# Neuroendocrine appendiceal tumor and endometriosis of the appendix: a case report

**DOI:** 10.1186/s13256-020-02490-x

**Published:** 2020-09-14

**Authors:** Rogério Serafim Parra, Marley Ribeiro Feitosa, Giovana Bachega Badiale Biagi, Daniel Ferracioli Brandão, Margarida Maria Fernandes da Silva Moraes, Liliane Silvestre, José Vitor Cabral Zanardi, Nelson Hitamo Sato Junior, Omar Féres, José Joaquim Ribeiro da Rocha

**Affiliations:** 1 Proctogastroclinic, Eliseu Guilherme St, 09, Ribeirão Preto, SP Brazil; 2 Department of Surgery and Anatomy, School of Medicine of Ribeirão Preto, University of São Paulo, Sao Paulo, Brazil; 3 M&M Laboratory of Pathology and Cytology, Ribeirão Preto, SP Brazil; 4 Nucleus - Diagnostic Medicine, SP Ribeirão Preto, Brazil; 5 Fecunditá Clinic, Ribeirão Preto, SP Brazil

**Keywords:** Appendiceal neoplasm, Appendix, Neuroendocrine tumor, Endometriosis, Deep infiltrating endometriosis

## Abstract

**Introduction:**

Endometriosis of the appendix is very uncommon, accounting for only about 1% of all cases of endometriosis. However, endometriosis is found in the appendix in approximately 8–13% of patients with deep infiltrating endometriosis and is particularly common in patients with severe forms of deep infiltrating endometriosis. Neuroendocrine tumors are the most common neoplasms of the appendix and may be misdiagnosed when there are multiple endometriosis lesions in the pelvis.

**Case presentation:**

We describe a case of a Caucasian patient with deep infiltrating endometriosis with rectal involvement, retrocervical lesions, and a right ovarian endometrioma with no suspected lesions in the appendix. She underwent laparoscopy and, after a systematic intraoperative evaluation, suspected involvement of the appendix was observed. The patient underwent ovarian cystectomy, excision of the pelvic endometriosis lesions, appendectomy, and anterior stapler discoid resection. Histopathological analysis of the appendix revealed endometriosis and a well-differentiated neuroendocrine carcinoma at the appendix tip.

**Discussion:**

Our patient’s case emphasizes the need to approach these lesions carefully and strengthens the indication for appendectomy when the appendix is affected in the setting of endometriosis. Despite the more likely diagnosis of appendiceal endometriosis, neuroendocrine tumors cannot be ruled out by imaging examinations, and both conditions can occur in the same patient.

## Introduction

Endometriosis is a common benign disease that affects approximately 10% of women of reproductive age and is associated with chronic pelvic pain, dyspareunia, and infertility [1]. Deep infiltrating endometriosis (DIE) is the most severe type and often affects the bowel (up to 25% of cases), particularly the rectosigmoid colon, and occasionally may be found in the ileum, cecum, and appendix [2, 3]. Surgery is indicated in patients with pelvic pain who do not respond to medical therapy and/or in patients with ileal involvement, owing to the risk of intestinal obstruction, and in those with appendiceal involvement, owing to the higher risk of neoplasia in these cases [2, 4]. Previous studies have suggested that appendiceal endometriosis is not a rare entity and occurs in up to 2.8% of patients with endometriosis [5] and up to 13.2% of patients with DIE [4]. The percentage is higher in patients with more severe disease or in those with lesions at multiple sites [4]. Therefore, systematic intraoperative evaluations of the appendix should be performed in patients with endometriosis, and in the presence of nodules, appendectomy must be considered [6, 7]. Neuroendocrine tumors are the most common neoplasms of the appendix and are detected in 0.16–2.3% of all appendectomies [8].

## Case presentation

**Fig. 1:**
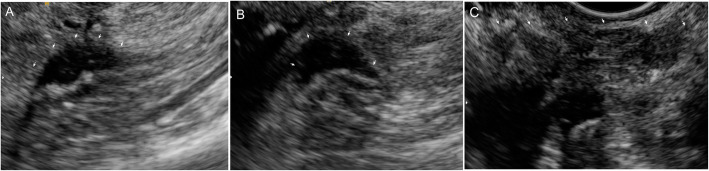
**a** Transvaginal ultrasound with bowel preparation: deep infiltrative endometriosis with rectal involvement, 9 cm of anal verge, 40% of circumference of rectum, 2.0 × 0.7 × 1.3 cm. **b** Retrocervical lesions. **c** 4.5-cm right ovarian endometrioma

**Fig. 2:**
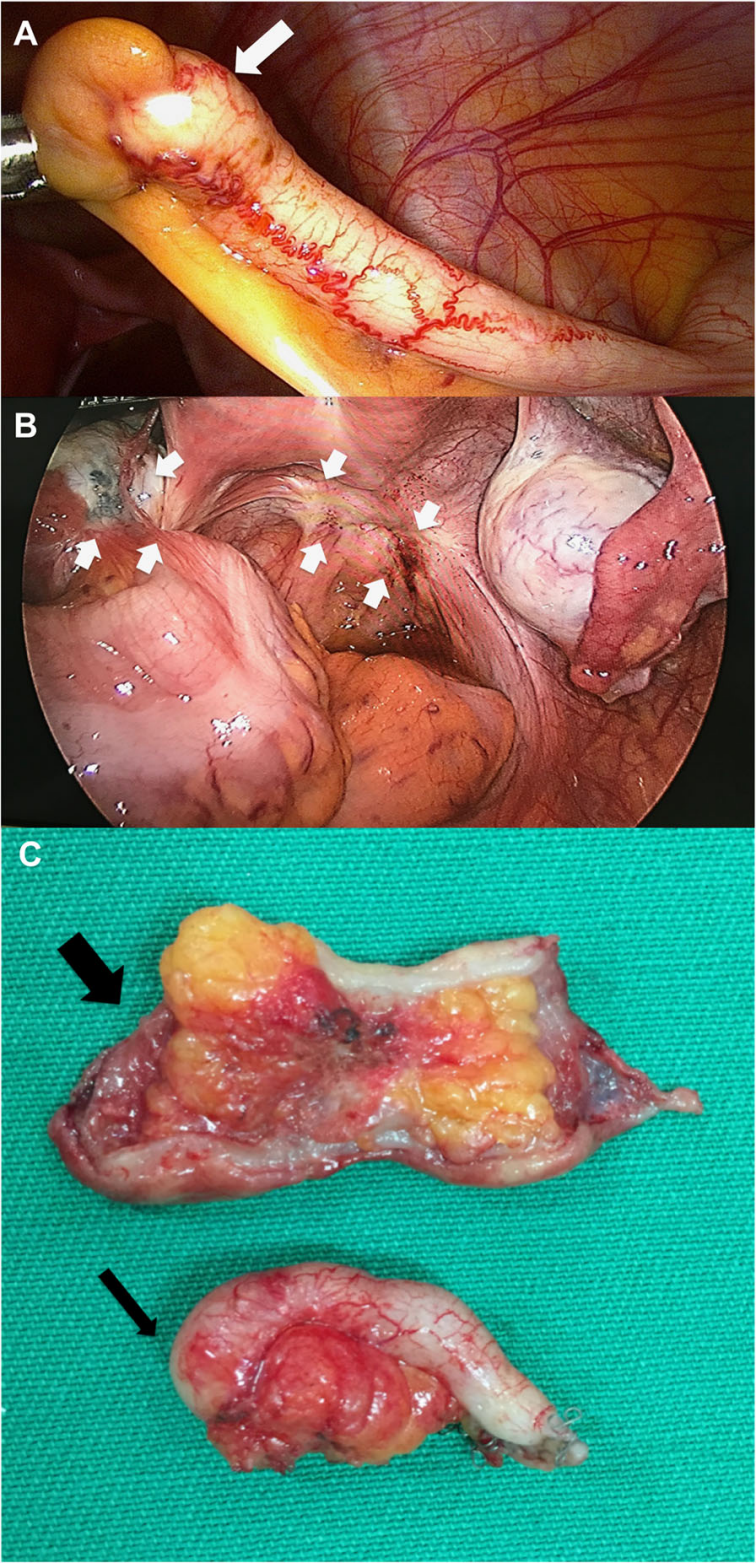
**a** Appendix with a nodular lesion at the tip (arrow). **b** Intraoperative evaluation of pelvic cavity showing (or revealing) endometriosis (arrows); **c** Surgical specimen (appendectomy - thin arrow; anterior stapler discoid resection - large arrow)

**Fig. 3:**
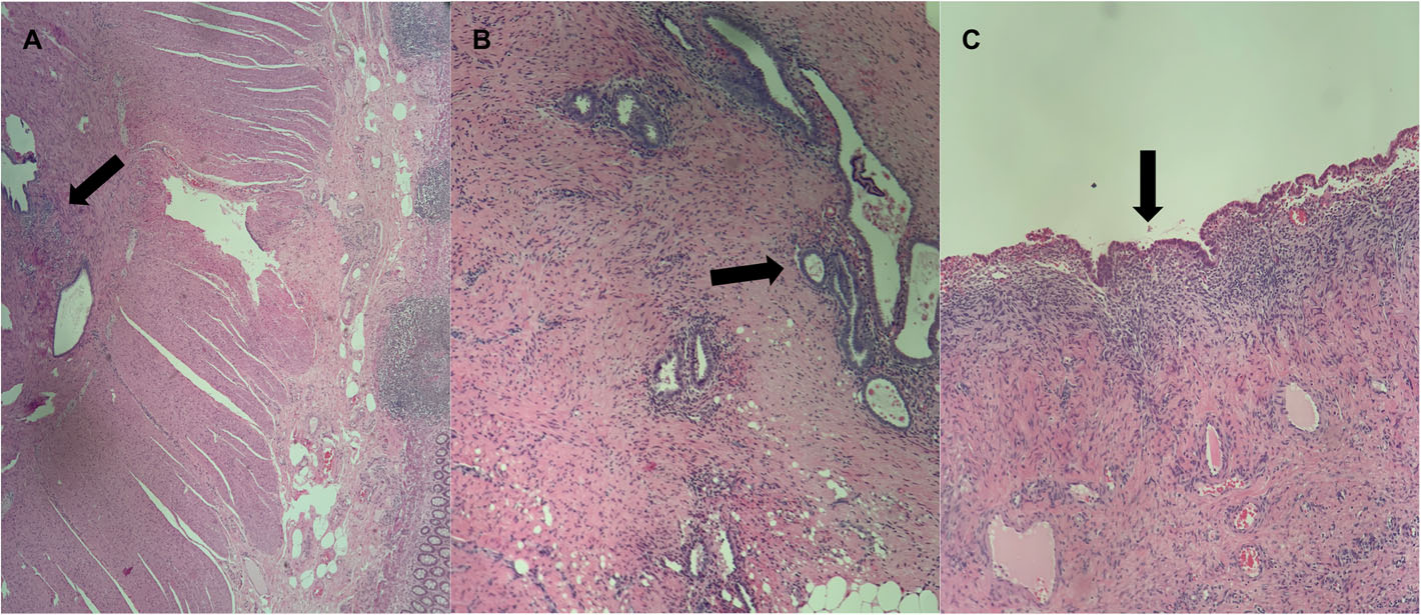
**a** and **b** Histopathological analysis of the rectum (arrow). **c** Histopathological analysis of the ovarian cystectomy (arrow). Both analyses confirmed extensive endometriosis involvement Hematoxylin and eosin (H&E) stain, original magnification 100 × and 400 ×)

**Fig. 4:**
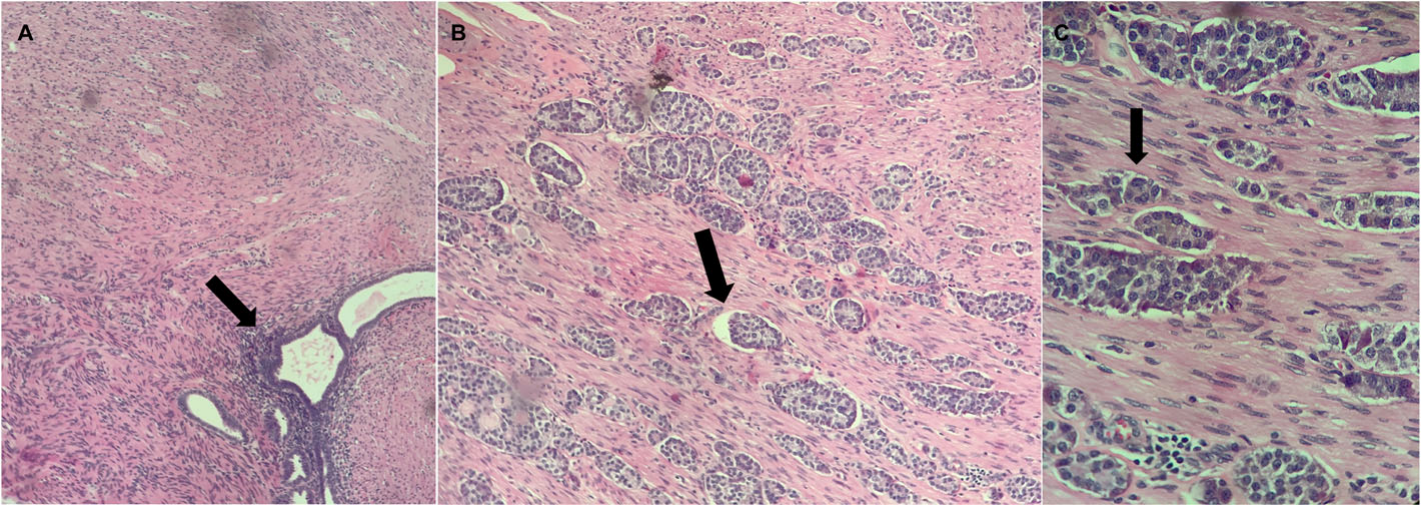
Histopathological analysis of the appendix. The analysis confirmed endometriosis (arrow) (**a**) and a well-differentiated neuroendocrine carcinoma infiltrating the adipose appendicular tissue, with angiolymphatic invasion and free surgical margins (arrow) (**b** and **c**) (Hematoxylin and eosin (H&E) stain, original magnification 100 × and 400 ×)

**Fig. 5:**
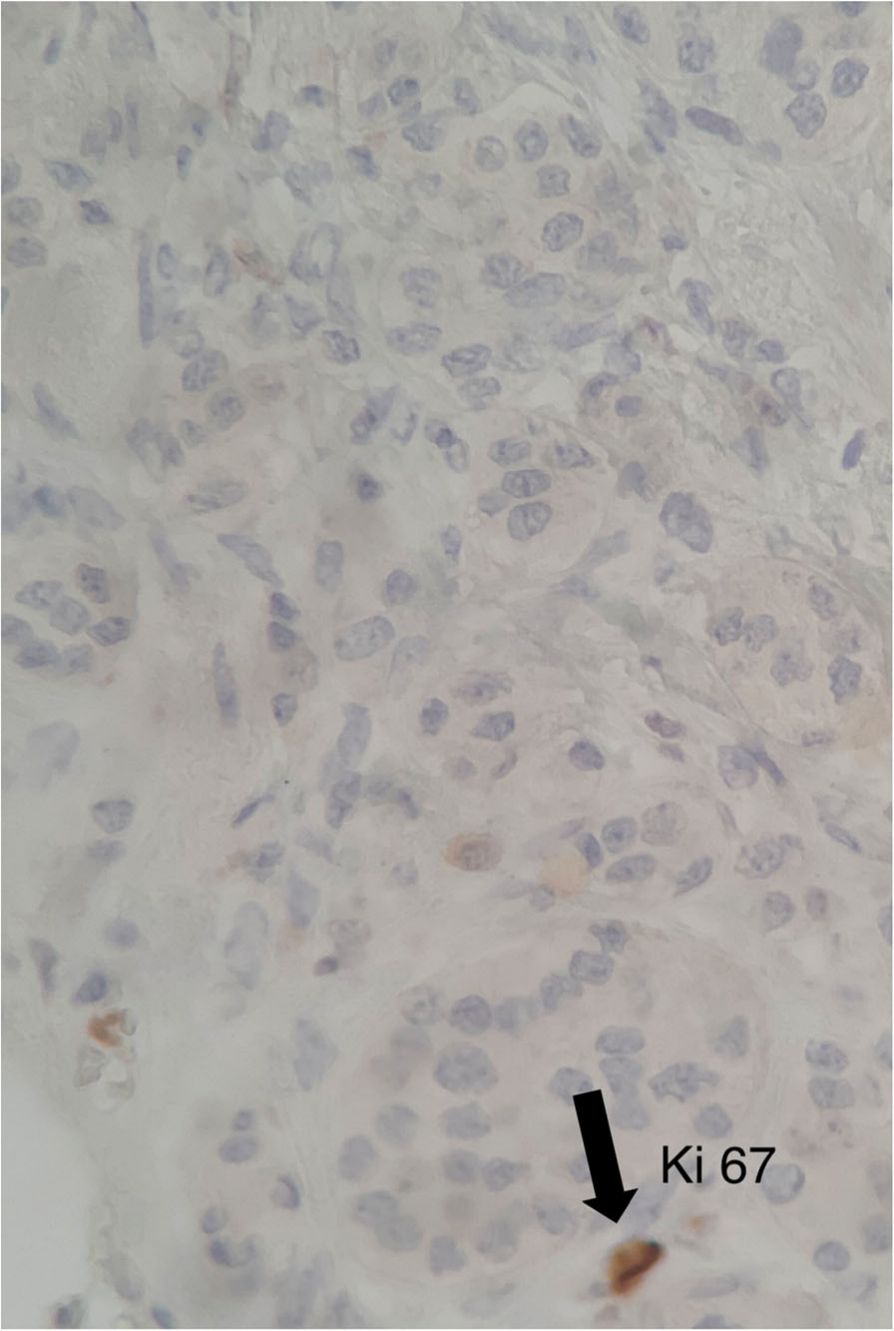
Immunohistochemical analysis. Positivity for Ki67/MIB-1 in 1.5% of cells (original magnification 400 ×)

A 45-year-old Caucasian woman was referred to our institution for deep dyspareunia, chronic pelvic pain, and dysmenorrhea. Her medical history revealed antidepressant treatment with no other family or personal history. She had previously undergone two failed *in vitro* fertilization treatments for infertility and had a history of previous treatments for endometriosis, including one laparoscopy. Her previous laparoscopy was performed in another institution (excision of superficial peritoneal nodules and uterosacral ligament). No bowel involvement was observed at first operation. Clinical examination showed absence of abdominal masses, mild pain on palpation of the lower abdomen, moderate pain on vaginal touch on uterine mobilization, and discomfort on rectal touch when the rectovaginal septum was palpated. Transvaginal ultrasound (TVU) (Fig. 1) with bowel preparation showed signs of deep endometriosis with rectal involvement (9 cm of anal verge, 40% of the circumference of the rectum, 2.0 × 0.7 × 1.3 cm), retrocervical lesions, and a 4.5-cm right ovarian endometrioma with no suspected lesions in the appendix. The patient did not undergo any other radiological imaging. She underwent laparoscopic surgery. A systematic intraoperative evaluation during laparoscopy revealed suspected involvement of the appendix with deep endometriosis. The patient then underwent ovarian cystectomy, excision of the pelvic endometriosis lesions, appendectomy, and anterior stapler discoid resection (Fig. 2). She was discharged in 1 day and had no postoperative complications. Histopathological analysis of the rectum and ovarian cystectomy confirmed extensive endometriosis involvement (Fig. 3), and the analysis of the appendix revealed endometriosis and a well-differentiated neuroendocrine carcinoma at the appendix tip that was 1.3 cm in size and infiltrated the adipose appendicular tissue with angiolymphatic invasion and free surgical margins (Fig. 4). Immunohistochemical analysis revealed positivity for Ki67/MIB-1 in 1.5% of the cells (Fig. 5). Right colectomy was indicated due to infiltration of the adipose tissue and due to angiolymphatic invasion, but the patient refused. She is currently being followed up, and, after 2 years, she has no signs of recurrence.

## Discussion

This study reinforces the importance of a complete evaluation of the peritoneal cavity, including the appendix, during laparoscopy for patients with DIE. In the presence of suspected lesions, appendectomy should always be performed. Previous studies demonstrated that endometriosis might be a cancer precursor; the mutations that are present in endometriosis-associated cancers can be found in adjacent endometriosis lesions [9].

It is important to state that the severity of patient symptomatology and disease state are not correlated, ranging from asymptomatic patients to patients with a myriad of complaints, including but not limited to dysmenorrhea, chronic pelvic pain, dyspareunia, infertility, fatigue, and cyclic urinary and intestinal symptoms according to the location of the disease. Lesions can be single or multifocal, including involvement of the appendix when endometriosis with intestinal involvement is present [10, 11].

Endometriosis of the appendix is very uncommon, accounting for only about 1% of all cases of endometriosis. However, in patients with endometriosis of the appendix, other sites are commonly affected by the disease, mainly the bladder and rectosigmoid and retrocervical regions. When these characteristics are present or if patients have more than three sites affected by endometriosis, the surgeon should evaluate the appendix carefully [12].

Our imaging protocol includes evaluation with TVS with a high-resolution linear transducer and bowel preparation in all patients to map the endometriosis lesions of the right iliac fossa and to detect lesions of the ileum, cecum, and appendix, as observed by other authors [6, 13]. Previous studies showed that ultrasonography has diagnostic accuracy not inferior to magnetic resonance imaging (MRI). The diagnostic performance of TVU and MRI are similar for detecting DIE involving the rectosigmoid colon, uterosacral ligaments, and rectovaginal septum. Therefore, it must be considered the primary approach for DIE diagnosis [14–16].

Appendectomy is mandatory when, during laparoscopic surgery for DIE, any abnormalities, such as the presence of nodules, thickening, swelling, or adhesions suggestive of disease in the appendix, are found, because the diagnosis of neuroendocrine tumor cannot be ruled out [6]. In addition [17, 18], a retrospective cohort study of patients with DIE with intestinal involvement was performed at our referral center from September 2014 to May 2019 (N = 124) [19] and showed appendiceal endometriosis in 12 patients (9.7%). Among all these patients, the presence of appendiceal neoplasia was detected in one patient, leading to a frequency of appendiceal neoplasia of 1 in 12 (8.3%) among all our appendectomies and 1 in 124 (0.8%) of all surgeries for DIE with intestinal resection. The incidence of appendectomies in our series is comparable with that in other series [4, 12], which reinforces the importance of a complete evaluation of the peritoneal cavity during laparoscopy in patients with DIE. In addition, it is essential to map the endometriosis lesions during the preoperative evaluation to facilitate surgical planning and ensure a complete evaluation of the intestine during laparoscopy.

Neuroendocrine tumors are detected in 0.16–2.3% of all appendectomies [8]. Generally, the prognosis is good, and the 5-year survival rate is higher than 95% [20]. Metastasis to regional lymph nodes and distant metastasis occur in approximately 4% and 1% of patients, respectively, usually when the primary tumor is larger than 2 cm. Appendectomy has been recommended as the treatment for appendiceal neuroendocrine tumors smaller than 1 cm in the guidelines set by the North American Neuroendocrine Tumor Society (NANETS). NANETS recommends right hemicolectomy in the following situations: tumors originating at the base of the appendix, tumors exceeding 2 cm in size, evidence of lymphovascular or mesoappendiceal invasion, lymph node metastasis, presence of regional lymph node metastasis, high mitotic count, peritoneal studding or angioinvasion, and intermediate or high-grade tumors [21]. If the proximal resection margin alone is involved, conservative local reexcision may be considered [6, 20].

In our patient’s case, we recommended a right colectomy to the patient, according to the recommendation described above. However, the patient refused to undergo another surgery. The colectomy was indicated by the histopathological results of angiolymphatic invasion. However, after follow-up of 24 months, the patient was free of recurrence. Recurrence has been tracked by imaging methods performed every 6 months.

## Conclusion

Our patient’s case emphasizes the need to approach these lesions carefully and strengthens the indication for appendectomy when the appendix is affected in the setting of endometriosis.

## Data Availability

The datasets supporting this article are included within the article.

## Abbreviations

DIE: Deep infiltrating endometriosis
MRI: Magnetic resonance imaging
NANETS: North American Neuroendocrine Tumor Society
